# Ambivalent Sexism, Social Roles, and Body Compassion in Albanian and Italian Women

**DOI:** 10.3390/bs16071245

**Published:** 2026-07-21

**Authors:** Cristian Di Gesto, Eriada Çela, Sonila Dubare, Amanda Nerini, Camilla Matera, Giulia Rosa Policardo

**Affiliations:** 1 Department of Psychology, Sapienza University of Rome, Via Dei Marsi, 78, 00185 Rome, Italy; 2 Department of Foreign Languages, Faculty of Human Sciences, University of Elbasan “Aleksandër Xhuvani”, Rruga “Ismail Zyma”, 3001 Elbasan, Albania; eriada.cela@uniel.edu.al; 3 Department of Epidemiology, College of Public Health and Health Professions, University of Florida, Gainesville, FL 32611, USA; soniladubare@ufl.edu; 4 Department of Education, Languages, Intercultures, Literatures and Psychology, University of Florence, 50135 Florence, Italy; amanda.nerini@unifi.it (A.N.); camilla.matera@unifi.it (C.M.); giuliarosa.policardo@uniecampus.it (G.R.P.)

**Keywords:** ambivalent sexism, social roles, body compassion, sociocultural contexts, women

## Abstract

This study investigated the relationships between ambivalent sexism, social roles, and body compassion in Albanian and Italian women. The participants were 251 Albanian and 280 Italian women who completed validated measures assessing hostile and benevolent sexism, social role transcendence, link to social roles, and three subdimensions of body compassion (defusion, common humanity, and acceptance). Path analyses indicated excellent model fit across samples. In Albanian women, hostile sexism negatively predicted social role transcendence and positively predicted a link to social roles, both of which were associated with lower body compassion. Benevolent sexism was positively associated with social role transcendence, which in turn was related to higher body compassion. In contrast, Italian women showed a different pattern: benevolent sexism positively predicted a link to social roles, while social role transcendence and link to social roles were both negatively related to defusion. Age positively predicted defusion and acceptance, highlighting a possible protective effect. Explained variance was higher in the Italian sample, particularly for the link to social roles. Overall, the findings suggest that the pattern of associations between sexist attitudes, social role attitudes, and body compassion was not identical across the two samples. These findings should be interpreted as exploratory evidence from two distinct sociocultural contexts rather than as formal cross-cultural comparisons.

## 1. Introduction

### 1.1. Body Image and Positive Body Image

The relationships between sociocultural factors and body image have been widely documented, particularly with regard to negative outcomes such as body dissatisfaction and appearance-related distress. However, considerably less attention has been devoted to understanding how these same forces relate to positive body image processes. In particular, body compassion represents a theoretically grounded construct that captures a kind, accepting, and non-judgmental stance toward one’s body, yet its sociocultural antecedents remain largely unexplored.

Building on objectification theory and sociocultural models of body image, this research examines the role of ambivalent sexism and social roles in shaping body compassion. Specifically, sexist ideologies are thought to reinforce traditional gender roles, which in turn may constrain women’s ability to relate to their bodies with acceptance, common humanity, and cognitive defusion. To investigate these mechanisms, the study examines two distinct sociocultural contexts, focusing on Italian and Albanian women, two contexts characterized by both shared cultural elements and distinct sociocultural trajectories.

Body image has long been conceptualized as a multidimensional construct that encompasses individuals’ perceptions, attitudes, and feelings toward their own bodies (Cash, 2005). For decades, empirical research has primarily emphasized its negative components, such as body dissatisfaction (Di Gesto et al., 2020; Nerini et al., 2024a; Roberts et al., 2022; Tylka & Kroon Van Diest, 2015), body shame, and appearance concerns (Di Gesto et al., 2025; Fardouly et al., 2018; Nerini et al., 2024b), which have been robustly associated with low self-esteem, depressive symptoms, and disordered eating (Rodgers et al., 2024; Stice, 2002; Thompson & Heinberg, 1999). This extensive body of work has significantly advanced our understanding of negative body image, yet it has also contributed to a predominantly deficit-oriented perspective, framing body image primarily as a site of vulnerability and pathology.

In response to this limitation, more recent scholarship has highlighted the need to expand the field toward positive body image, focusing on protective and adaptive processes that foster resilience in the face of appearance-related pressures (Matera et al., 2024; Tylka & Wood-Barcalow, 2015). Positive body image is not simply the absence of dissatisfaction, but rather reflects appreciation, acceptance, and respect for the body (Tylka & Wood-Barcalow, 2015; Wood-Barcalow et al., 2024). Whereas constructs such as body satisfaction primarily describe evaluative outcomes related to one’s body, body compassion has been conceptualized as an integrative, process-oriented construct that describes how individuals respond to body-related experiences and difficulties with acceptance, cognitive defusion, and recognition of common humanity, consistent with mindfulness- and acceptance-oriented approaches (e.g., ACT). Conceptually, it comprises three interrelated processes—defusion, common humanity, and acceptance—that support adaptive responding to appearance- and body-related challenges (Altman et al., 2020). Defusion refers to stepping back from body-related thoughts and emotions, treating them as transient mental events rather than literal truths, thereby reducing over-identification with perceived imperfections. Common humanity entails recognizing that concerns and frustrations about the body are shared human experiences rather than isolating personal failings. Acceptance denotes a kind, non-judgmental willingness to be with painful body-related cognitions and feelings, responding to them with self-kindness rather than self-criticism. Together, these processes articulate a compassionate way of relating to the body that aligns with contemporary positive body-image frameworks and has been linked to lower body dissatisfaction and higher psychological well-being in prior work (Altman et al., 2020; Policardo et al., 2021). Body compassion thus reflects a compassionate stance toward one’s body, countering critical or perfectionistic tendencies that often arise in sociocultural contexts emphasizing appearance.

Emerging evidence suggests that body compassion serves a protective role, buffering against the harmful effects of beauty societal standards (de Carvalho Barreto et al., 2020; Oliveira et al., 2018; Policardo et al., 2025). Higher levels of body compassion have been linked to reduced body shame, lower levels of appearance anxiety, and increased well-being (Oliveira et al., 2018). By promoting acceptance and kindness toward one’s appearance, body compassion provides an avenue through which women may resist internalizing restrictive appearance norms. Despite its growing relevance, relatively little is known about the sociocultural and attitudinal antecedents of body compassion. Drawing on Objectification Theory (Fredrickson & Roberts, 1997), sexist ideologies and traditional gender-role prescriptions may undermine women’s capacity to relate to their bodies with acceptance, common humanity, and cognitive defusion. Accordingly, examining these sociocultural variables may contribute to a better understanding of the processes that foster or hinder body compassion as a specific process-oriented component of positive body image (Frederick & Reynolds, 2022).

### 1.2. Social Roles and Body Image

Social roles can be defined as widely shared cultural beliefs about the attributes and responsibilities considered appropriate for women and men. According to Social Role Theory (Eagly & Wood, 2012), such stereotypes arise from the historical division of labor and become institutionalized as prescriptive norms: women are typically linked to communal and caregiving responsibilities, whereas men are tied to agentic qualities such as assertiveness and leadership (Eagly & Kite, 1987; Eagly & Wood, 2012). Although often framed as merely descriptive, these beliefs are deeply normative, rewarding conformity and penalizing deviation. Their pervasiveness has been demonstrated cross-culturally, with robust attributions of communal traits to women and agentic traits to men across diverse societies (Williams & Best, 1982; Williams et al., 1999). Psychological and sociological frameworks converge in explaining how such expectations take root in the self: gender schema theory describes how cultural definitions of masculinity and femininity organize self-concepts (Bem, 1981); gender role strain highlights the psychological costs of rigid adherence to prescriptive roles (Levant, 2011; Pleck, 1981, 1995); and sociological perspectives conceptualize gender as performance and as an order of power relations, underscoring the institutionalization of these scripts (Connell, 2009; Goffman, 1977). In traditionalist systems, certain behaviors, roles, and careers are stereotypically coded as “masculine” or “feminine”; masculinity (instrumentality) is associated with assertiveness, competitiveness, independence, and aggressiveness, whereas femininity (expressiveness) emphasizes emotionality, care, and group harmony (Constantinople, 1973; Spence & Helmreich, 1980). Empirical evidence has shown that women tend to self-describe with culturally feminine traits more than men, whereas men more often endorse stereotypically masculine traits (Mueller & Dato-On, 2008; Spence et al., 1975).

These role prescriptions intersect with body image in consequential ways. From a sociocultural perspective, appearance-related expectations are woven into role mandates, reinforcing the notion that women’s social value is tied not only to caregiving or communal behavior, but also to physical attractiveness (Fredrickson & Roberts, 1997). In this vein, studies show that stronger endorsement of traditional role expectations is associated with greater internalization of beauty ideals and higher vulnerability to body dissatisfaction (Cash et al., 1997; Forbes et al., 2006; Jackson et al., 1988; Swami et al., 2008). Related work links gender-role orientation to adverse health correlates for women, including body dissatisfaction and eating-disorder risk (Murnen & Smolak, 1997; Swami & Abbasnejad, 2010). Conversely, stereotype transcendence—moving beyond prescriptive roles—has been associated with greater autonomy and resistance to appearance pressures (Beere, 1990), suggesting that role beliefs function as sociocultural inputs that might shape how women relate to their bodies, either amplifying appearance anxiety and body dissatisfaction or supporting acceptance and resilience.

Taken together, this literature suggests that social role stereotypes are closely intertwined with body image and may shape women’s body-related experiences through broader ideological systems. Because these role beliefs are frequently legitimized and reinforced by ambivalent sexism, examining sexism and social roles within a single theoretical framework may help clarify the sociocultural processes underlying body compassion.

### 1.3. Ambivalent Sexism and Body Image

*Ambivalent Sexism Theory* (Glick & Fiske, 1996, 2001) conceptualizes sexism as a multidimensional system composed of hostile sexism—overtly antagonistic attitudes toward women who challenge male dominance—and benevolent sexism—subjectively positive but paternalistic beliefs that idealize women in subordinate, dependency-oriented roles. Although they differ in tone, the two components are mutually reinforcing: hostile sexism punishes deviation from gender prescriptions, whereas benevolent sexism rewards conformity by casting subordination as desirable and protective. Accordingly, research shows that ambivalent sexism is tied to stronger endorsement of traditional role expectations and resistance to gender equality (Sibley et al., 2007a). Feminist analyses since the 1970s further argue that these ideologies position women’s bodies as central to social worth, embedding appearance norms within patriarchal power relations (Patil, 2013).

Empirical work links ambivalent sexism to body image-relevant beliefs and behaviors. Some studies indicate that higher ambivalent sexism relates to greater thin ideal internalization (Forbes et al., 2007) and more favourable evaluations of appearance-enhancing practices (Swami et al., 2010). In a recent study with Chinese university students, hostile sexism was positively associated with acceptance of cosmetic surgery, with gender-role attitudes mediating this association, highlighting how sexist ideologies can operate through social role beliefs to shape appearance orientations (Zhang et al., 2024). Complementary lines of evidence grounded in objectification theory show that objectifying experiences and self-surveillance are associated with greater consideration or acceptance of cosmetic procedures (Calogero et al., 2010); moreover, work on subtle/modern sexism suggests that women who have undergone such procedures may report higher implicit sexist attitudes (Eriksen & Goering, 2011; Swim et al., 1995). Taken together, these findings suggest that ambivalent sexism legitimizes appearance norms directly and also indirectly by reinforcing traditional social-role prescriptions.

Crucially, research to date has been predominantly oriented toward negative indicators of body image (e.g., body dissatisfaction). While this focus has clarified key risk pathways, it leaves open important questions about how hostile and benevolent sexism, together with social roles, relate to protective processes such as body compassion. Addressing this gap can clarify whether the same attitudinal forces that sustain gendered role expectations also undermine women’s capacities for defusion, recognition of common humanity, and acceptance in the face of body-related challenges.

### 1.4. The Current Study

Two key gaps emerge from the existing literature. First, although a substantial body of research has linked ambivalent sexism and social roles to negative body-related outcomes—including body dissatisfaction, appearance anxiety, and acceptance of cosmetic surgery (Cash et al., 1997; Forbes et al., 2006, 2007; Jackson et al., 1988; Swami et al., 2008, 2010; Tiggemann & Kuring, 2004; Zhang et al., 2024)—no research has examined their association with positive body image. Constructs such as body compassion represent important resources for understanding how women may develop resilience in the face of sociocultural pressures, yet their relationship with sexism and stereotyped social role remains unexplored. This limits a comprehensive understanding of how gendered ideologies may not only undermine women’s relationships with their bodies but also constrain the development of protective processes.

Second, existing studies have been predominantly conducted within single cultural contexts, leaving unclear whether the associations between sexism, social roles, and body image are universal or context-specific. Examining these associations in distinct sociocultural contexts may provide insight into whether the proposed theoretical model is similarly or differentially expressed across countries.

Italy and Albania represent particularly informative contexts for such an investigation, not only because of their geographical proximity and shared Mediterranean influences, but also due to their distinct sociocultural and historical trajectories, which may differentially shape gender ideologies and body-related experiences. Italy has undergone processes of modernization and gender equality reforms within a Western European framework, while simultaneously maintaining a strong cultural emphasis on beauty ideals and gendered expectations (Dotti Sani & Quaranta, 2017). This coexistence of progressive norms and persistent appearance-related pressures makes Italy a context in which ambivalent sexism and social role expectations may operate in more subtle and socially normalized forms.

Albania, conversely, has experienced rapid social and economic transitions following decades of communist rule, where traditional gender roles have persisted alongside emerging influences of globalization and European integration (Abazi & Doja, 2017). Similar to other Eastern European countries, the communist regime rhetorically promoted gender equality through women’s participation in the workforce and universal access to education, while simultaneously maintaining patriarchal structures, traditional family roles, and limited individual autonomy (Gal & Kligman, 2000). The post-communist period has been characterized by a renegotiation of values, in which traditional norms have often regained prominence, potentially reinforcing more explicit and prescriptive gender-role expectations.

These differences are theoretically relevant for the current study, as variations in the strength, form, and social acceptability of gender-role prescriptions and sexist ideologies may influence how women relate to their bodies. In particular, contexts characterized by stronger or more rigid role expectations may be less conducive to the development of body compassion, as they may reinforce evaluative and appearance-based self-relations. Examining Italy and Albania side by side therefore provides a meaningful opportunity to explore whether the associations between ambivalent sexism, social roles, and body compassion are consistent across contexts or vary as a function of broader sociocultural dynamics.

To address these gaps, this study investigates the relationships between ambivalent sexism, social roles, and body compassion in two sociocultural contexts: Albanian and Italian women. Drawing on the literature reviewed above, we hypothesized that hostile and benevolent sexism would be indirectly associated with lower body compassion across its three dimensions (defusion, common humanity, and acceptance), through lower social roles transcendence and stronger adherence to traditional social roles (modeled as parallel mediators). Given the exploratory nature of the comparison between the two samples, no a priori differences between the two samples were assumed; instead, potential variations were examined descriptively.

From this perspective, the present study should not be interpreted as a formal cross-cultural comparison of latent constructs. The primary objective was to estimate the proposed theoretical model separately within the Italian and Albanian samples and to explore whether the pattern of associations among ambivalent sexism, social role attitudes, and body compassion appeared conceptually similar or different across the two sociocultural contexts. Accordingly, any observed between-country differences are interpreted as exploratory and descriptive rather than as evidence of psychometrically equivalent cross-cultural differences. Moreover, given the use of different language versions of the instruments across samples, formal measurement invariance could not be established, and therefore stronger cross-cultural conclusions cannot be drawn. Accordingly, the present findings should be interpreted as preliminary evidence of associations within two independent samples rather than as evidence of psychometrically equivalent cross-cultural differences.

Overall, this study extends previous research by examining body compassion as a positive body image outcome and by testing the indirect associations between ambivalent sexism, social roles, and body compassion in two different cultural contexts through parallel mediation models.

## 2. Materials and Methods

### 2.1. Participants

The study included a total of 531 women (N = 531; M = 32.2, SD = 8.44), comprising 251 Albanian women (M = 32.2, SD = 8.34) and 280 Italian women (M = 32.2, SD = 8.53).

Considering that the questionnaire was administered in English to the Albanian subsample, they were also asked to self-evaluate their English language proficiency across four domains. Regarding understanding, 62.0% reported a very good level, 20.0% good, 13.0% fair, and smaller proportions indicated a poor (3.0%) or very poor (1.0%) level. For speaking skills, 51.0% declared a very good level, 23.0% good, 21.0% fair, 4.0% poor, and 1.0% very poor. With respect to reading, 65.0% reported very good competence, 18.0% good, 13.0% fair, 3.0% poor, and 1.0% very poor. Finally, for writing in English, 55.0% considered their level very good, 22.0% good, 18.0% fair, 4.0% poor, and 1.0% very poor. These indices indicate generally high self-reported proficiency, reducing the likelihood that limited language skills systematically biased responses in the Albanian sample.

A detailed distribution of participants’ sociodemographic characteristics by country is presented in Table 1.

### 2.2. Measures

Sociodemographic details. Sociodemographic information (i.e., age, nationality, place of birth, place of residence, marital status, education, occupation) was collected in both the Italian and Albanian subsamples.

Ambivalent sexism. Levels of hostile and benevolent sexism were assessed using the Ambivalent Sexism Inventory (ASI; Glick & Fiske, 1996). For the Italian sample, the validated Italian version was employed (Manganelli Rattazzi et al., 2008), whereas for the Albanian sample, the original English version of the scale was administered. This scale measures individuals’ endorsement of sexist attitudes, distinguishing between hostile and benevolent sexism. The hostile sexism subscale consists of 11 items (e.g., “Feminists are making unreasonable demands of men”), while the benevolent sexism subscale also includes 11 items (e.g., “Women should be cherished and protected by men”). Items were rated on a 6-point Likert scale ranging from 0 (Strongly disagree) to 5 (Strongly agree). Higher scores on each subscale indicate greater levels of hostile or benevolent sexism, respectively. Internal consistency was α = 0.89, ω = 0.90 for hostile sexism and α = 0.90, ω = 0.90 for benevolent sexism in the Italian sample, and α = 0.88, ω = 0.89 for hostile sexism and α = 0.89, ω = 0.89 for benevolent sexism in the Albanian sample.

Social roles. Gender role attitudes were assessed using the *Social Roles Questionnaire* (SRQ; Baber & Tucker, 2006). For the Albanian sample, the original English version of the instrument was employed, whereas for the Italian sample we used an Italian version used in previous studies (e.g., Tortora et al., 2020). The SRQ is composed of 13 items measuring two dimensions of gender role attitudes: Social Role Transcendence (5 items; e.g., “People should be treated the same regardless of their sex”), which reflects egalitarian views on gender roles, and Social Role Link (8 items; e.g., “Only some types of work are appropriate for both men and women”), which reflects the belief that certain roles are inherently linked to gender. Participants rated each item on a 6-point Likert scale ranging from 0 (Strongly disagree) to 5 (Strongly agree). Higher scores on each subscale indicate greater levels of transcendent or traditional views of social roles, respectively. Since the SRQ had not previously been validated in the Italian context, we tested its factorial structure through confirmatory factor analysis (CFA) and assessed its reliability in terms of internal consistency using both Cronbach’s alpha (α) and McDonald’s omega (ω). CFA supported the bifactorial model of the Italian version with a good fit to the data (χ^2^ = 71.4, *p* < 0.01; χ^2^/df = 1.74; RMSEA = 0.06; CFI = 0.97). Reliability indices were satisfactory in both samples. For the Italian sample, internal consistency was α = 0.89, ω = 0.89 for Social Role Transcendence, and α = 0.90, ω = 0.90 for Social Role Link. For the Albanian sample, internal consistency was α = 0.88, ω = 0.88 for Social Role Transcendence, and α = 0.89, ω = 0.89 for Social Role Link.

Body compassion. Body compassion was assessed using the Body Compassion Scale (BCS; Altman et al., 2020). For the Italian sample, the validated Italian version was employed (Policardo et al., 2021), whereas for the Albanian sample, the original English version of the scale was administered. The BCS is composed of 23 items that measure compassionate attitudes toward one’s body and comprises three subscales: defusion (9 reverse-coded items; e.g., “When I feel frustrated with my body’s inability to do something, I tend to feel separate and cut off from other people”), common humanity (9 items; e.g., “When I am frustrated with some aspect of my appearance, I try to remind myself most people feel this way at some time”), and acceptance (5 items; e.g., “I am accepting of my looks just the way they are”). Before completing the scale, participants were instructed to respond based on how they generally felt about their body. Items were rated on a 5-point Likert scale ranging from 1 (Almost never) to 5 (Almost always). Higher scores on each subscale indicate greater levels of body defusion, common humanity, and body acceptance, respectively. Internal consistency was excellent in both samples: in the Italian sample, α = 0.89, ω = 0.89 for defusion, α = 0.88, ω = 0.88 for common humanity, and α = 0.88, ω = 0.88 for acceptance; in the Albanian sample, α = 0.88, ω = 0.88 for defusion, α = 0.89, ω = 0.89 for common humanity, and α = 0.89, ω = 0.90 for acceptance.

### 2.3. Procedure

Recruitment was conducted through social media outreach (e.g., LinkedIn, Facebook, X, Instagram, WhatsApp groups) and collaborations with local institutions, community networks, and university departments, using a direct and opportunistic sampling approach in both Italy and Albania. Eligibility criteria required participants to (a) be 18 years or older and (b) have sufficient proficiency in the language of administration to independently complete the survey. However, no strict cut-off threshold was applied based on self-reported levels. Instead, participants were included provided they indicated at least a basic ability to understand the questionnaire content. These procedures were adopted to balance inclusivity with the need to ensure adequate understanding of the measures. To enhance comparability across samples, similar recruitment strategies were adopted in both countries, primarily relying on online outreach and collaborations with community networks. Although some contextual differences in recruitment channels and the use of different language versions of the instruments were unavoidable, efforts were made to ensure that data collection procedures were as consistent as possible across the two samples.

For the Italian sample, the survey was administered in Italian. For the Albanian sample, the original English versions of the measures were employed. During the pilot phase with Albanian students, participants were asked to evaluate the clarity and comprehensibility of the questionnaire items and to indicate whether any wording required modification. These pilot tests indicated that the content was understandable and culturally appropriate, and no changes to the original items were deemed necessary; all instruments were administered in their original form.

Participants were informed that their participation was voluntary, anonymous, and confidential, and that they could withdraw from the study at any time without justification. Data were collected through an online questionnaire administered via the Qualtrics^®^ platform (Provo, UT, USA, 2020) (Qualtrics, 2020). The introductory page outlined the study’s objectives, inclusion criteria, confidentiality safeguards, and researcher contact details. Only individuals who confirmed their eligibility and provided informed consent were granted access to the survey, which began with socio-demographic questions followed by self-report measures. No financial or material compensation was provided.

Completion of the questionnaire required approximately 15 min. The study was approved by the Ethics Committee of the University of Florence (Prot. No. 0148845 of 5 July 2023, Minute No. 259 of 3 July 2023). All procedures adhered to the ethical standards of the institutional and/or national research committee, as well as the 2024 Helsinki Declaration principles and guidelines.

### 2.4. Data Analyses

All statistical analyses were conducted using Jamovi software (The Jamovi Project, 2023; version 2.4.8.0). First, descriptive statistics and *r* Pearson’s correlations among all study variables—ambivalent sexism (hostile and benevolent sexism), social roles (social role transcendence and social role link), and body compassion (defusion, common humanity, and acceptance)—were computed to examine their distributions and interrelationships.

Second, we examined the fit of three path analysis models in which hostile sexism and benevolent sexism were posited as antecedents of (a) social role transcendence, (b) link to social roles, and (c) the three dimensions of body compassion (i.e., defusion, common humanity, and acceptance), respectively. These models were tested separately for the two subsamples, resulting in three models for the Italian sample and three models for the Albanian sample. Based on preliminary correlations, age was included as a control variable only in the Italian subsample, as correlational analyses showed that age was not significantly associated with any of the main study variables in the Albanian sample (all *r* < 0.10, *p* > 0.05). Education level was also examined as a potential covariate because of the observed differences in educational attainment between the two samples. Supplementary analyses including education level yielded virtually identical results, with no meaningful changes in the magnitude, direction, or statistical significance of the estimated parameters. Therefore, in accordance with the principle of model parsimony, the more parsimonious models are presented in the manuscript. Social role transcendence and link to social roles were allowed to covary. All the assumptions for path analysis were satisfied (Streiner, 2005). Because the models were estimated independently within each sample, the analyses were intended to examine within-group associations rather than to perform formal cross-cultural comparisons of latent constructs.

The hypotheses were tested using Jamovi (The Jamovi Project, 2023; version 2.4.8.0). Bootstrapping procedures were applied to estimate the indirect effects and their size, thereby testing mediation (Rucker et al., 2011). The overall sample size in this study exceeded the recommended minimum of 200 participants for structural models (Weston & Gore, 2006). Parameter estimates were derived using the maximum likelihood procedure.

Model fit was evaluated using multiple indices: the χ^2^/df ratio, for which values of 2 or below indicate good fit (Tabachnick & Fidell, 2007); the Comparative Fit Index (CFI) and the Tucker–Lewis Index (TLI), with values ≥ 0.95 reflecting excellent fit (Hu & Bentler, 1999); the Goodness-of-Fit Index (GFI), with values ≥ 0.95 indicating good fit (Hooper et al., 2008); the Non-Normed Fit Index/Relative Noncentrality Index (NNFI/RNI), with values ≥ 0.95 considered acceptable (Bentler & Bonett, 1980); the Root Mean Square Error of Approximation (RMSEA) along with its 90% confidence interval, with values ≤ 0.06 considered good fit (Hu & Bentler, 1999); and the Standardized Root Mean Square Residual (SRMR), with values ≤ 0.08 acceptable (Hooper et al., 2008).

## 3. Results

### 3.1. Descriptive Statistics and Bivariate Correlation

Table 2 reports the descriptive statistics (means and standard deviations) and bivariate correlations among the study variables in Italian and Albanian women. Normality was assessed using the Shapiro–Wilk test in combination with inspection of skewness and kurtosis values. Although the Shapiro–Wilk test indicated some deviations from normality, this result was expected given the relatively large sample size and the sensitivity of the test to minor distributional departures. Inspection of skewness (−1.68 to 1.85) and kurtosis (−0.51 to 6.24) suggested no severe violations of normality. Overall, the distributional properties were considered acceptable for the purposes of the analyses conducted.

Considering the descriptive statistics, mean age and BMI were similar in both samples. Within the psychosocial variables, the Albanian sample was characterized by higher mean scores for hostile sexism, benevolent sexism, and link to social roles, whereas the Italian sample was characterized by higher mean scores for social role transcendence. Regarding body compassion, the Italian sample showed higher mean scores for defusion and common humanity, whereas the Albanian sample showed higher mean scores for acceptance. These descriptive differences are presented to characterize the two samples and should not be interpreted as formal between-sample comparisons.

The correlational analyses revealed distinct patterns within each sample. In the Italian sample, age was positively associated with defusion and acceptance, while it was negatively associated with common humanity. BMI did not show significant correlations with any of the study variables. Hostile sexism was positively correlated with benevolent sexism and link to social roles, and negatively correlated with social role transcendence. Benevolent sexism was positively associated with link to social roles and negatively associated with social role transcendence, defusion, common humanity, and acceptance. Social role transcendence was negatively associated with defusion and positively associated with common humanity. Link to social roles was negatively correlated with defusion, common humanity, and acceptance. Defusion was positively associated with common humanity and acceptance, whereas common humanity was positively correlated with acceptance.

In the Albanian sample, neither age nor BMI showed significant associations with the study variables. Hostile sexism was positively correlated with benevolent sexism and link to social roles, while being negatively associated with defusion. Benevolent sexism was positively associated with social role transcendence, link to social roles, and common humanity, and negatively associated with defusion. Social role transcendence was positively correlated with link to social roles, common humanity, and acceptance. Link to social roles was negatively associated with defusion. Defusion was negatively associated with common humanity, whereas common humanity was positively correlated with acceptance.

### 3.2. Path Models

Six path models were estimated—one for each of the three body compassion dimensions (defusion, common humanity, acceptance) in the Italian and Albanian subsamples. All models exhibited excellent global fit. In the Italian subsample, model fit indices ranged from χ^2^ = 7.39 to 8.66 with df = 6 and corresponding *p* values between 0.19 and 0.29, yielding χ^2^/df ratios of 1.23–1.44. RMSEA was consistently 0.04, with 90% confidence intervals ranging from [0.00, 0.09] to [0.00, 0.10]. SRMR values fell between 0.02 and 0.03, and all comparative fit measures (CFI, TLI, RNI, GFI) were equal to 0.98–0.99. Comparable fit indices were observed in the Albanian subsample, with χ^2^ values ranging from 2.36 to 3.02, df = 2, *p* = 0.22–0.31, χ^2^/df = 1.18–1.51, RMSEA = 0.03–0.05 with 90% confidence intervals ranging from [0.00, 0.13] to [0.00, 0.15], SRMR = 0.01–0.02, and all additional fit indices equal to 0.99.

Across the six independently estimated models, the pattern of associations between sexism and the two mediators was largely consistent within the two samples. In both samples, hostile sexism was negatively associated with social roles transcendence and positively associated with link to social roles. Benevolent sexism was positively associated with link to social roles only in the Italian sample, whereas in the Albanian sample it was positively associated with social roles transcendence. This shared structural pattern provided a useful framework for interpreting the associations observed across the three body compassion dimensions within each sample.

#### 3.2.1. Defusion

**Figure 1:**
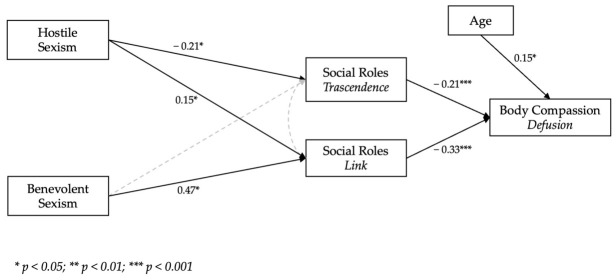
Observed model *Defusion* (Italian sample).

**Figure 2:**
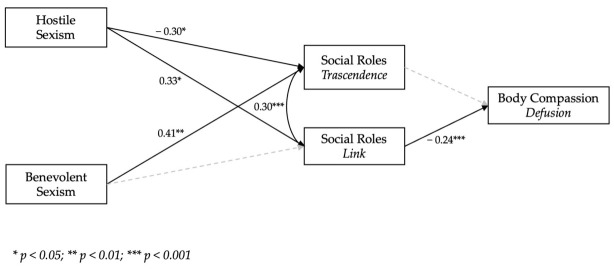
Observed model *Defusion* (Albanian sample).

For defusion, in the Italian sample (Figure 1), social roles transcendence positively predicted defusion, whereas link to social roles showed a negative association. Bias-corrected bootstrapping indicated significant indirect effects through link to social roles for both hostile sexism (indirect = −0.070, 95% CI [−0.141, −0.010]) and benevolent sexism (indirect = −0.219, 95% CI [−0.354, −0.111]). The indirect effect via transcendence was positive and small but significant for hostile sexism (0.058, 95% CI [0.010, 0.121]) and non-significant for benevolent sexism (−0.004, 95% CI [−0.060, 0.047]). The model accounted for 17% of the variance. In the Albanian sample (Figure 2), link to social roles negatively predicted defusion, whereas social roles transcendence did not show a significant association. Bootstrapped confidence intervals supported a significant negative indirect effect via link for hostile sexism (−0.073, 95% CI [−0.169, −0.006]), while all other indirect effects included zero (hostile via transcendence: −0.026, 95% CI [−0.075, 0.013]; benevolent via link to social roles: −0.048, 95% CI [−0.122, 0.006]; benevolent via social role transcendence: 0.035, 95% CI [−0.015, 0.095]). The model explained 15% of the variance in defusion.

#### 3.2.2. Common Humanity

**Figure 3:**
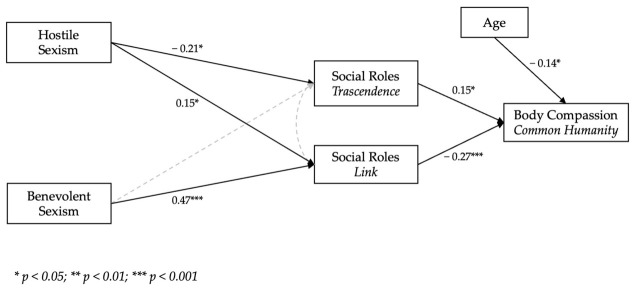
Observed model *Common Humanity* (Italian sample).

**Figure 4:**
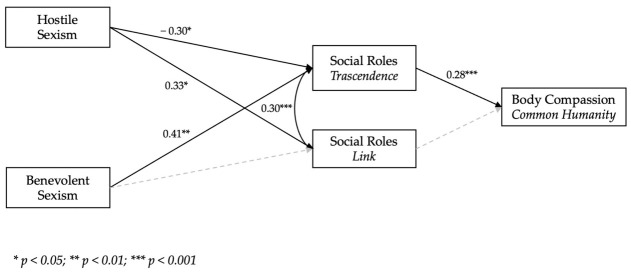
Observed model *Common Humanity* (Albanian sample).

For common humanity, in the Italian sample (Figure 3), social roles transcendence was positively associated with the outcome, whereas link to social roles showed a negative association. Bootstrapped analyses revealed significant negative indirect effects through link to social roles for both hostile sexism (−0.055, 95% CI [−0.109, −0.004]) and benevolent sexism (−0.174, 95% CI [−0.267, −0.091]). Indirect effects via social roles transcendence were small and significant for hostile sexism (−0.041, 95% CI [−0.105, −0.002]) and non-significant for benevolent sexism (0.003, 95% CI [−0.040, 0.044]). The model explained 12% of the variance. In the Albanian sample (Figure 4), social roles transcendence positively predicted common humanity, whereas the path from link to social roles was not significant. Bootstrapped indirect effects via social roles transcendence were significant for benevolent sexism (0.115, 95% CI [0.035, 0.236]) and hostile sexism (−0.086, 95% CI [−0.187, −0.014]), while all indirect paths via link to social roles included zero. The model explained 18% of variance.

#### 3.2.3. Acceptance

**Figure 5:**
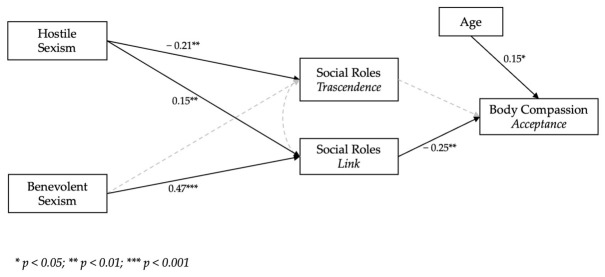
Observed model *Acceptance* (Italian sample).

**Figure 6:**
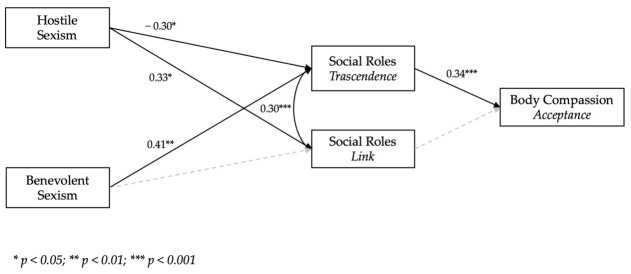
Observed model *Acceptance* (Albanian sample).

For acceptance, in the Italian subsample (Figure 5), link to social roles negatively predicted the outcome, whereas social roles transcendence did not. Significant negative indirect effects via link to social roles were observed for hostile sexism (−0.060, 95% CI [−0.120, −0.007]) and benevolent sexism (−0.190, 95% CI [−0.308, −0.082]); indirect effects via transcendence included zero. The model explained 10% of the variance. In the Albanian subsample (Figure 6), social roles transcendence positively predicted acceptance, whereas link to social roles did not. Bootstrapped analyses supported significant indirect effects via social roles transcendence for benevolent sexism (0.134, 95% CI [0.039, 0.251]) and hostile sexism (−0.101, 95% CI [−0.212, −0.014]), while indirect effects via link to social roles were non-significant. The model accounted for 11% of variance.

## 4. Discussion

The present study examined how ambivalent sexism and social role stereotypes relate to the components of body compassion—defusion, common humanity, and acceptance—in two distinct sociocultural contexts represented by Italian and Albanian women. Across both samples, the findings show that sexism is indirectly associated with lower body compassion, but the psychological mechanisms linking these constructs appear to vary across the two sociocultural contexts represented in the present samples. Although the observed differences are discussed within the broader sociocultural literature on gender roles and sexism, it is important to emphasize that the current study did not directly assess cultural values, gender norms, or related contextual variables. Accordingly, the present findings should be interpreted as evidence of within-sample associations rather than as formal evidence of cross-cultural differences between latent constructs.

A first consistent pattern across models was the connection between sexist attitudes and social role beliefs. Hostile sexism was associated with lower social role transcendence and a stronger link to traditional social roles in both samples; benevolent sexism was correlated with a link to social roles in Italy and with transcendence in Albania. These associations accord with Ambivalent Sexism Theory, which describes a coordinated system in which overt hostility polices departures from prescriptive femininity and paternalistic “warmth” rewards conformity (Glick & Fiske, 1996, 2001), and with Social Role Theory, which explains how the historical division of labor becomes normative guidance for self and behavior (Eagly & Kite, 1987; Eagly & Wood, 2012). It also resonates with research showing that sexist ideologies travel with stronger endorsement of traditional gender roles and resistance to equality-promoting change (Sibley et al., 2007b).

Against this general backdrop, the three dimensions of body compassion warrant separate consideration. In what follows, we discuss defusion, common humanity, and acceptance in turn, attending to how the social roles-based pathways manifest within each cultural setting and whether they converge or diverge between the Italian and Albanian models.

### 4.1. Defusion

In Italy, link to social roles was associated with lower defusion, and both hostile and benevolent sexism were indirectly related to reduced defusion through this pathway. This configuration is consistent with sociocultural and objectification accounts in which prescriptive femininity positions appearance as a salient standard of evaluation, sustaining self-surveillance and limiting the space for cognitive distance from evaluative body thoughts (Cash et al., 1997; Fredrickson & Roberts, 1997; Jackson et al., 1988). Social role transcendence was also negatively associated with defusion. From a gender role strain perspective, resisting entrenched scripts can entail social friction and cognitive load, especially where beauty norms remain salient, so moving away from prescriptive roles does not necessarily translate into immediate decentering from body-related judgments (Pleck, 1981, 1995). The small indirect association linking higher hostile sexism to higher defusion via reduced transcendence should be interpreted cautiously, as it likely reflects a complex pattern in which hostile sexist beliefs constrain role flexibility while body-related cognitive distance may also be shaped by other unmeasured factors.

In Albania, the link to social roles was again associated with lower defusion, and hostile sexism showed an indirect association with reduced defusion through this pathway, reflecting its relation to stronger social roles endorsement. One possible interpretation is that this pattern may reflect a sociocultural context in which prescriptive obligations keep attention anchored to how one “should” look to meet role expectations, thereby undermining cognitive distance from body-focused thoughts (Eagly & Kite, 1987; Eagly & Wood, 2012; Fredrickson & Roberts, 1997). However, because contextual variables related to gender norms were not directly measured, this interpretation should be regarded as a theoretically informed hypothesis rather than a direct conclusion from the data. In contrast, social role transcendence did not display an association with defusion, indicating that questioning role prescriptions may be a necessary but insufficient condition for decentering when beauty norms continue to function as social capital (Bordo, 2013; Hesse-Biber, 2007). Benevolent sexism did not show reliable indirect effects on defusion; the pattern of associations with the mediators appears insufficient in strength and coherence to translate into an effect on the outcome.

### 4.2. Common Humanity

In the Italian models, social role transcendence was positively associated with common humanity, whereas the link to social roles was negatively associated with it. In line with this pattern, hostile and benevolent sexism were associated with lower common humanity primarily through stronger endorsement of traditional social roles. Consistent with objectification theory and sociocultural accounts, prescriptive femininity treats appearance and propriety as central indices of personal worth; where such standards are emphasized, women are encouraged to monitor themselves against ideals, and body difficulties are more likely to be construed as personal shortcomings rather than as experiences shared with others (Cash et al., 1997; Fredrickson & Roberts, 1997). Viewed through the lenses of Ambivalent Sexism Theory and Social Role Theory (Eagly & Kite, 1987; Eagly & Wood, 2012; Glick & Fiske, 1996, 2001), the small indirect path is compatible with the idea that hostile sexist beliefs relate to stricter role prescriptions, and that less flexibility in role beliefs may make a shared-humanity stance more difficult when confronting appearance demands.

In Albania, social role transcendence was again positively related to common humanity, whereas the path from link to social roles was not significant. Here, hostile sexism was connected to lower common humanity through reduced transcendence, while benevolent sexism was related to higher common humanity through greater transcendence. These findings may suggest that moving beyond prescriptive roles is associated with a greater tendency to reframe body difficulties as part of the shared human experience (Altman et al., 2020; Beere, 1990). It also aligns with work showing that sexist ideologies operate through role beliefs in shaping appearance-related orientations (Forbes et al., 2007; Swami et al., 2010; Zhang et al., 2024). The positive path from benevolent sexism through greater social role transcendence does not indicate that benevolent sexism is protective. In contexts where roles are being renegotiated, paternalistic messages that appear affirming may coexist with increased flexibility in role definition and enactment of roles, which can support common humanity, while still subtly reinforcing traditional expectations. However, since the current study did not directly assess cultural beliefs or gender-role norms, this interpretation remains speculative and should be interpreted cautiously.

### 4.3. Acceptance

The Italian results for acceptance were consistent with the defusion and common-humanity models on the social roles belief variables. Endorsing traditional social roles related to lower acceptance of difficult body-related experiences, and both hostile and benevolent sexism were indirectly connected to lower acceptance through this endorsement. This configuration fits sociocultural and objectification accounts in which prescriptive femininity keeps appearance and propriety under continual evaluation, making a receptive, non-judgmental stance toward bodily sensations harder to sustain (Cash et al., 1997; Fredrickson & Roberts, 1997). It is also aligned with acceptance-based models of positive body image, which hold that self-kindness toward the body is undermined when women are chronically compared with prescriptive standards (Altman et al., 2020). By contrast, social role transcendence did not carry a significant pathway to acceptance in this sample. Within a Social Role Theory lens, distancing from prescriptive roles may not, by itself, loosen appearance-focused self-evaluation enough to foster a compassionate stance; when role expectations remain salient in everyday contexts, acceptance may depend more on the degree to which those expectations are affirmed than on abstract distancing from them (Eagly & Kite, 1987; Eagly & Wood, 2012). The positive association of age with acceptance is consistent with work suggesting that, over time, women often report lower investment in appearance and more tolerant responses to bodily change, possibly due to shifts in life priorities and accumulated coping resources (Slevec & Tiggemann, 2010).

In Albania, patterns were centered on social role transcendence rather than on a link to social roles. Transcendence was positively associated with acceptance, whereas the link to social roles did not show a significant association. Higher hostile sexism co-occurred with lower acceptance through its association with lower transcendence, whereas higher benevolent sexism co-occurred with higher acceptance through its association with greater transcendence. Read alongside Ambivalent Sexism Theory and Social Role Theory, these results may suggest that flexibility in role beliefs represents one possible mechanism associated with a more receptive, non-judgmental response to body-related discomfort in this context (Eagly & Kite, 1987; Glick & Fiske, 1996, 2001). Where women can step outside prescriptive expectations, the space for acceptance appears to widen, cohering with conceptualizations of body compassion as an attitude of openness and kindness in the face of bodily distress (Maas genannt Bermpohl et al., 2023). The positive path involving benevolent sexism should not be interpreted as indicating that benevolent sexism is protective. One possible explanation is that, in some sociocultural contexts, paternalistic statements may be perceived as expressions of care rather than exclusively as mechanisms of social control. This interpretation may be particularly relevant in transitional societies such as Albania, where traditional gender norms continue to coexist with ongoing social and cultural change. In these contexts, benevolent sexism may not only reinforce conventional gender expectations but may also be experienced as a source of social approval, relational security, and affirmation of culturally valued feminine identities. Such dynamics may facilitate a more accepting relationship with one’s body, not because benevolent sexism is psychologically beneficial per se, but because conformity to prevailing gender expectations may provide a sense of belonging and social validation. In turn, this social validation may temporarily reinforce a coherent sense of feminine identity that is culturally valued, thereby reducing the immediate impact of appearance-related concerns. In this sense, conformity to prevailing gender expectations may temporarily function as a psychosocial buffer against appearance-related self-evaluation, although this buffering effect is likely to depend on the persistence of traditional normative systems and should not be interpreted as evidence of long-term psychological benefit. If so, endorsement of benevolent sexism may coexist with relational orientations that are associated with greater acceptance (Sibley et al., 2007b; Swami et al., 2010; Szymanski et al., 2009). Nevertheless, because these cultural meanings were not directly assessed in this study, this interpretation should be considered hypothetical and requires empirical verification.

These findings also warrant a broader consideration regarding the interpretation of the positive indirect associations involving benevolent sexism observed in some models, particularly within the Albanian sample. Although these patterns may appear counterintuitive, they should not be interpreted as evidence of a protective function of benevolent sexism. Rather, the following interpretations should be viewed as theoretically plausible explanations rather than empirical demonstrations, and several non-mutually exclusive mechanisms may account for these findings.

This interpretation is consistent with sociological accounts describing post-socialist societies such as Albania as contexts in which traditional and egalitarian gender norms often coexist during periods of social transition. From this perspective, benevolent sexism may become more deeply embedded in normative belief systems and therefore less likely to be experienced as overtly restrictive. Under such conditions, endorsement of benevolent sexist beliefs may coexist with greater flexibility in role interpretation, allowing for the emergence of adaptive self-relational processes such as common humanity or acceptance, without implying a genuine challenge to gender prescriptions.

At the same time, these associations may reflect response tendencies shaped by cultural norms emphasizing relational harmony, conformity, and socially desirable responding. In such contexts, agreement with benevolent sexist items may partially capture interpersonal orientation rather than strict ideological endorsement, thereby inflating associations with constructs linked to acceptance and tolerance.

Lastly, methodological considerations should also be taken into account. The reliance on self-report measures and the use of English-language instruments in the Albanian sample may have contributed to these patterns. Collectively, these factors suggest that the observed indirect effects should be interpreted with caution and understood as preliminary. Future research is needed to disentangle whether these findings reflect culturally specific meanings of gender roles, response styles, or measurement-related artifacts, rather than substantive psychological processes.

### 4.4. Summary of Key Findings

Taken together, the six models support the central hypothesis: ambivalent sexism relates to lower body compassion primarily through social role stereotypes. In Italy, the link to social roles emerges as the principal conduit undermining all three components of body compassion, consistent with contexts in which appearance norms are tightly interwoven with communal expectations and everyday standards of propriety (Cash et al., 1997; Fredrickson & Roberts, 1997). In Albania, social role transcendence plays a more prominent supportive role for common humanity and acceptance, whereas the link to social roles most clearly undermines defusion. Across both cultural contexts, the findings converge with work showing that sexist ideologies are tied to thin ideal internalization and favorable orientations toward appearance regulation, often via role beliefs (Forbes et al., 2007; Swami et al., 2010; Zhang et al., 2024). Nevertheless, these differences should not be interpreted as direct evidence of specific cultural processes, since this study did not include direct measures of cultural values or contextual gender norms. Future cross-cultural research should explicitly examine these constructs to test the theoretical interpretations proposed here.

The results also extend positive body image theory by showing that defusion, common humanity, and acceptance are not uniformly affected by the same social role mechanisms. Defusion seems particularly sensitive to the pull of social role obligations, which encourage evaluative thinking and self-surveillance; common humanity and acceptance appear to flourish where role transcendence is viable and socially supported. Placed alongside Ambivalent Sexism Theory, Social Role Theory, and objectification accounts (Eagly & Kite, 1987; Fredrickson & Roberts, 1997; Glick & Fiske, 1996, 2001), this pattern suggests that cultivating body compassion depends not only on individual psychological skills but also on the ideological climate—the degree to which femininity is tethered to role compliance and attractiveness.

In summary, sexist attitudes aligned with social role beliefs, and those beliefs were implicated in women’s capacity to engage with their bodies through cognitive distance from harsh self-evaluations, recognition of shared humanity, and a kind, accepting stance. Where prescriptive social roles were strongly endorsed, body compassion was diminished; where stepping beyond prescriptions was possible, sharedness and acceptance were more likely to take root. This integrated account helps explain why protective processes are unequally distributed across sociocultural contexts and clarifies the pathways through which gendered ideologies shape women’s embodied lives.

This study offers several contributions. First, it broadens the scope of body image research by extending work on positive body image and treating body compassion—defusion, common humanity, and acceptance—as distinct and theoretically relevant outcomes. Much prior work has emphasized maladaptive endpoints; positioning protective processes at the core of the analyses advances theory on how women can cope adaptively with appearance-related pressures (Altman et al., 2020; Policardo et al., 2021). Second, the models clarify the role of social role stereotypes in linking sexist ideologies to body compassion by integrating complementary frameworks—Ambivalent Sexism Theory, Social Role Theory, and Objectification Theory—into a single explanatory pathway (Eagly & Kite, 1987; Fredrickson & Roberts, 1997; Glick & Fiske, 1996, 2001). This specification highlights how links to social roles and social role transcendence operate concurrently rather than as mutually exclusive alternatives. Third, the study provides a cross-cultural comparison of women in Italy and Albania, revealing that similar ideological aspects can assemble differently across settings; distinguishing the roles of social roles transcendence and link to social roles clarifies why some components of body compassion are more vulnerable in one context than another. Finally, the parallel-mediator specification offers a more differentiated account of process, showing that links to social roles and social role transcendence operate concurrently rather than as mutually exclusive alternatives.

Despite its contributions, this study has several limitations that should be acknowledged. First, the cross-sectional design prevents any causal inferences about the directionality linking ambivalent sexism, social role stereotypes, and body compassion. Longitudinal and experimental designs would be needed to examine whether changes in sexist attitudes or role beliefs prospectively influence defusion, common humanity, or acceptance. Second, all variables were assessed using self-report measures, which may introduce common-method bias and social desirability concerns. Third, both samples were self-selected and recruited through non-probabilistic online snowball sampling, which limits the generalizability of the findings to the broader populations of Italian and Albanian women. Moreover, participants in both samples were predominantly highly educated, suggesting that women with lower educational attainment and those from rural or less digitally connected settings may have been underrepresented. Accordingly, the findings should be interpreted as reflecting the characteristics of this specific sample rather than being fully representative of the broader female populations in the two countries. Additionally, some sociodemographic differences between the Italian and Albanian samples (e.g., education and employment status) should be considered when interpreting the findings. These differences may reflect both broader structural characteristics of the respective national contexts and sample-specific recruitment dynamics. Given the study design, it is not possible to disentangle these sources of variation, and therefore cross-cultural comparisons should be interpreted with caution. Fourth, the use of English-language questionnaires in the Albanian sample, in the absence of formally validated and culturally adapted versions of the instruments, represents a methodological limitation that may have implications for semantic equivalence and response accuracy. Although participants reported generally high levels of English proficiency across comprehension, speaking, reading, and writing, self-reported language competence cannot fully substitute for formal linguistic validation. This choice was driven by the current lack of validated instruments in the Albanian language and was made in collaboration with the local research team to ensure feasibility and contextual appropriateness; however, future research should prioritize the development and validation of culturally and linguistically adapted measures in this context. This asymmetry in language administration across samples may have further implications for cross-cultural comparability, as formal measurement invariance could not be established. Consequently, the present findings should be interpreted as exploratory evidence of associations within two independent cultural samples rather than as definitive evidence of cross-cultural differences in the underlying latent constructs. Future studies should establish configural, metric, and scalar invariance before conducting formal comparisons across cultures.

Importantly, while a confirmatory factor analysis (CFA) was conducted in the Italian sample to verify the expected structure within that context, the factor structure was not formally tested in the Albanian sample. Accordingly, measurement equivalence across groups cannot be assumed. However, consistent with the exploratory nature of the study, the primary focus was on examining patterns of associations within each cultural group rather than establishing cross-cultural measurement invariance or making strong between-group comparisons. Future research should extend this work by implementing full cross-cultural validation procedures, including translation, back-translation, and psychometric testing (e.g., EFA/CFA) in independent samples.

Another limitation concerns the mediating variables examined in the present study. The study was specifically designed to investigate the role of social role attitudes in the association between ambivalent sexism and body compassion. Consequently, other theoretically relevant mechanisms proposed in the literature, such as body surveillance, body shame, and the internalization of appearance ideals, were beyond the scope of the present study and were therefore not assessed. Future research should examine these complementary pathways within more comprehensive theoretical models.

Moreover, the study did not assess or statistically control for several potentially relevant sociocultural and contextual factors, such as media exposure, gender norms within local communities, or other cultural influences, which may have affected the observed associations. Future research should incorporate these variables into more comprehensive models to better isolate the specific contribution of ambivalent sexism and social role attitudes to body compassion.

A final limitation concerns the interpretation of the observed cross-cultural differences. Although the findings were discussed in light of existing sociocultural theories, the present study did not directly assess cultural values, gender ideologies, or broader contextual characteristics. Consequently, the cultural explanations proposed should be regarded as theoretically grounded hypotheses rather than empirically verified mechanisms. Future studies should incorporate direct measures of these constructs to better understand the processes underlying cross-cultural variation and to empirically test the theoretical interpretations advanced in this study.

## 5. Conclusions

Across two national samples, this study examined how ambivalent sexism relates to body compassion—defusion, common humanity, and acceptance—through social role stereotypes specified as social role transcendence and the link to social roles. The six models converged on the same pattern: hostile and benevolent sexism were associated with role beliefs, which in turn shaped women’s capacity to meet body-related challenges with psychological distance, a sense of shared humanity, and an accepting stance. In Italy, a stronger link to social roles consistently undermined all components of body compassion; in Albania, social role transcendence more strongly supported common humanity and acceptance, while the link to social roles chiefly reduced defusion. These configurations indicate that similar ideological variables assemble differently across sociocultural contexts.

Conceptually, the findings place body compassion within a sociocultural pathway connecting ambivalent sexism, social role stereotypes, and compassionate responding to the body, consistent with Ambivalent Sexism Theory, Social Role Theory, and objectification accounts. Practically, the results suggest that reducing both hostile and benevolent sexism, fostering more flexible role beliefs, and strengthening skills related to defusion, common humanity, and acceptance may help cultivate protective processes, with culturally responsive adaptations depending on whether link to social roles or social role transcendence is the more influential pathway.

In sum, this work provides an initial cross-cultural framework illustrating how ambivalent sexism, role beliefs, and body compassion interrelate in everyday life, offering concise guidance for culturally sensitive prevention and intervention efforts aimed at supporting positive body image.

## Figures and Tables

**Table 1:** Sociodemographic characteristics of participants by country.

Variable	Category	Italy (%)	Albania (%)
Age	Italian	32.2, SD = 8.53	32.2 (SD = 8.34)
	Albanian		
Nationality	Italian	100.0	–
	Albanian	–	100.0
Marital Status	Single	49.3	39.8
	Married/Cohabiting	47.9	49.8
	Separated/Divorced	2.9	10.4
	Other	–	–
Education	Lower secondary	2.1	–
	Upper secondary	23.2	3.2
	BA degree	39.3	17.5
	MA/Old system degree	35.4	79.3
	Other	–	–
Occupation	Full-time	22.5	67.3
	Part-time	26.4	7.2
	Occasional	17.9	0.8
	Seeking first job	5.7	4.0
	Student	26.1	15.9
	Unemployed	1.4	4.8

**Table 2:** Descriptive statistics (M, SD) and bivariate correlations among study variables in Italian (*n* = 280) and Albanian (*n* = 251) women.

Variable	Italy M (SD)	Albania M (SD)	1	2	3	4	5	6	7	8	9
1. Age	32.20 (8.53)	32.20 (8.34)	-	0.06	−0.05	−0.05	−0.09	−0.06	0.02	−0.07	−0.05
2. BMI.	21.90 (2.99)	21.95 (3.19)	−0.02	-	0.05	0.05	0.04	0.04	0.07	−0.11	−0.03
3. Hostile Sexism	1.66 (0.70)	1.80 (1.10)	−0.01	0.02	-	0.89 ***	0.07	0.53 ***	−0.13 *	0.07	0.08
4. Benevolent Sexism	1.15 (0.67)	1.99 (1.14)	−0.02	0.06	0.78 ***	-	0.14 *	0.52 ***	−0.16 **	0.12 *	0.11
5. SRQ—Transcendence	7.93 (2.46)	6.90 (3.20)	−0.04	−0.06	−0.20 ***	−0.15 *	-	0.30 ***	0.02	0.29 ***	0.33 ***
6. SRQ-Link	1.29 (1.51)	2.78 (2.30)	0.10	−0.01	0.53 ***	0.59 ***	−0.04	-	−0.21 ***	0.09	0.04
7. BCS-Defusion	3.88 (0.94)	3.75 (1.01)	0.12 *	0.06	−0.11	−0.21 ***	−0.20 ***	−0.31 ***	-	−0.29 ***	0.11
8. BCS-Common Humanity	3.58 (0.93)	3.22 (1.13)	−0.17 **	0.03	−0.11	−0.18 **	0.16 **	−0.29 ***	0.24 ***	-	0.41 ***
9. BCS-Acceptance	3.75 (1.06)	3.95 (1.08)	0.12 *	0.04	−0.09	−0.16 **	0.01	−0.24 ***	0.65 ***	0.57 ***	-

Note. Correlations below the diagonal = Italian sample; correlations above the diagonal = Albanian sample. BMI: Body Mass Index; SRQ: Social Role Questionnaire; BCS: Body Compassion Scale. Asterisks indicate the statistical significance of the Pearson correlation coefficients: * *p* < 0.05; ** *p* < 0.01; *** *p* < 0.001.

## Data Availability

The data are not publicly available but are held by the corresponding author and can be provided upon reasonable request and justified purpose. The dataset is fully anonymous.
